# Tumor-produced immune regulatory factors as a therapeutic target in cancer treatment

**DOI:** 10.3389/fimmu.2024.1416458

**Published:** 2024-08-14

**Authors:** Vladimir Rogovskii

**Affiliations:** Department of Molecular Pharmacology and Radiobiology, Pirogov Russian National Research Medical University, Moscow, Russia

**Keywords:** cancer, inflammation, cytokines, immune-regulatory factors, immune suppression

## Introduction

For hundreds of years, mankind has attempted to fight cancer by directly destroying tumor cells utilizing various cytotoxic substances. However, such strategies have generally failed to produce lasting success, especially when it comes to metastatic tumors. More specific approaches, such as targeted therapies and immunotherapy, hold more promise (1–4). Here we will discuss another approach to cancer therapy - targeting the production of factors released by tumor cells. Although it may be impossible to directly kill all tumor cells in an effective and durable manner while maintaining an acceptable safety profile, it is at least might be possible to reduce the production of factors by tumor cells that cause immunosuppression (5–8).

Nowadays, studies mainly are not focused on suppression of tumor-produced cytokines and other factors, despite the notion that they play important role in cancer-induced immune suppression (9–11). The continuous production of various cytokines may be not less important for the spread of cancer than the proliferation of cancer cells. This cytokine production generates a kind of immunosuppressive cocktail that causes local immune unresponsiveness to cancer antigens and serves as a source of autocrine growth factors for cancer cells (9, 12, 13).

For instance, conditioned media from human tumor-derived cells isolated from cancer tissue of treatment-naive patients with melanoma or ovarian cancer prominently induced dendritic cell dysfunction (11). Conditioned media from pancreatic cancer cells and pancreatic stellate cells induced differentiation of myeloid-derived suppressor cells (MDSCs) and suppression of lymphocytes (14). Conditioned media derived from lung cancer cells induced pro-tumoral phenotypes in macrophages (15). Stimulation of B cells with breast cancer cell-conditioned media caused the development of regulatory B cells (Breg) contributing to tumor evasion from the immune response (16, 17).

Of note, each factor in the tumor microenvironment has a dual role. They can stimulate anticancer immunity or act as tumor promoters and induce negative feedback in immune regulation. This “dual role” is commonly described in scientific literature, in discussions of cytokines such as the IL-1 family (18), IL-6 (19), TNF (20), IL-10 (21), and others. Many sources indicate that, in the tumor microenvironment, tumor-derived factors mainly play a tumor-promoting role. Therefore, targeting them may be an effective strategy in fighting cancer (9, 11, 22–25).

**Figure 1:**
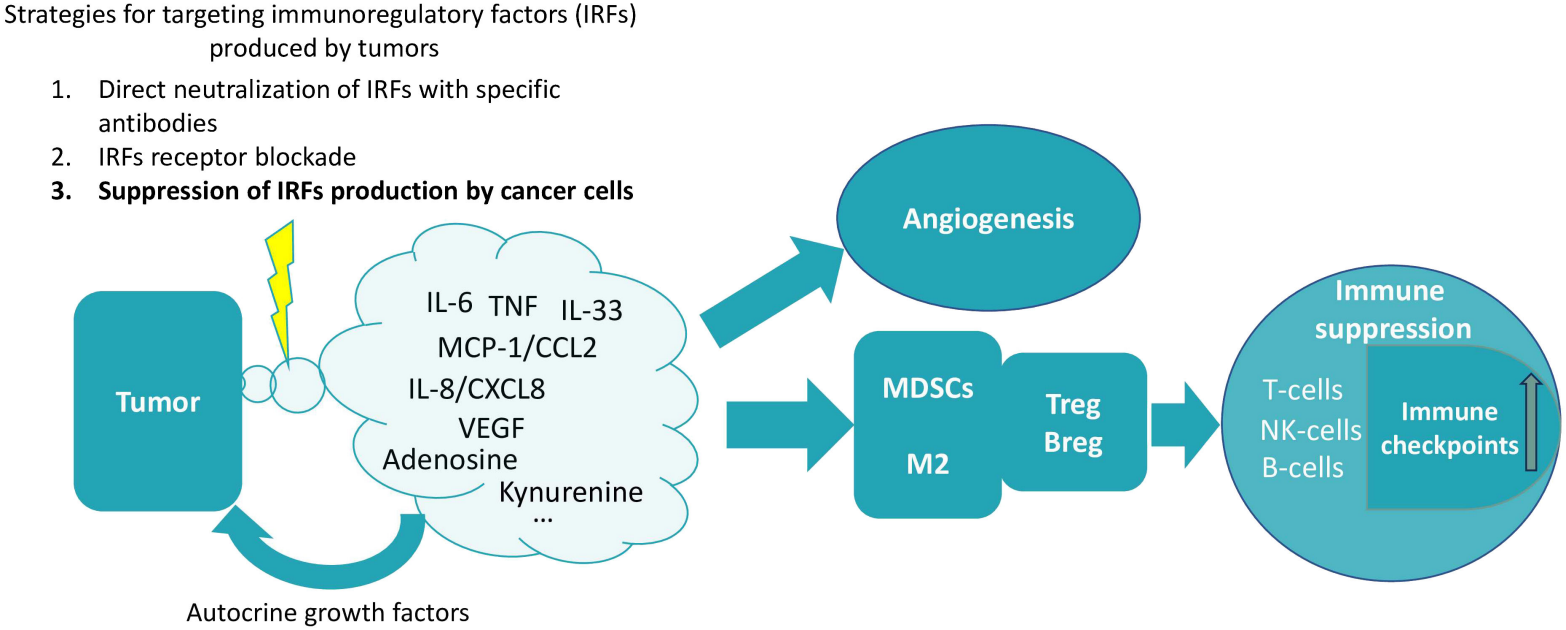
The production of various soluble factors that induce immunosuppression is a characteristic feature of tumor cells. Cancer cells can serve as an example of the diseased tissue. Such tissues are characterized by the production of proinflammatory cytokines and chemokines (28). Normally, such production of immune-regulatory factors is dependent on the microenvironment. However, in cancer cells, cytokine production is unconditional and context-independent, as cells of different cancer types are capable of spontaneous cytokine production (29–31). A pattern can be identified in which these factors cause the recruitment of various myeloid cells into the tumor tissue, which acquire an immunosuppressive phenotype in the tumor microenvironment, triggering a chain of further suppression of immune functions. In particular, under their influence, the expansion of T- and B-regulatory cells occurs and the expression of various immune checkpoints increases (17, 32–35). Reducing the production of these factors by tumor cells may therefore be a promising strategy for cancer therapy. It is extremely important to note that tumors do not produce a single factor, but rather a whole set of factors of different types (different depending on the type of tumor). This plethora of factors produced by tumors contributes to their perception as a network structure, in which it is difficult to single out one major factor (6, 36). Therefore, for therapeutic purposes, it seems that the totality of these factors (or at least some of them) should be targeted at once. The influence on angiogenesis is an example of the perception of tumor-produced factors as components of a network structure, where there are many mutual influences (37). In particular, not only VEGF affects angiogenesis, but also many of the other factors are capable of angiogenesis upregulation [for instance, IL-6 (38), TNF (39), IL-33 (40), MCP-1/CCL2 (41), IL-8/CXCL10 (42), kynurenine (43), adenosine (44)].

The paradox is that despite the role of immunity in defense against cancer, immune cells are also required for carcinogenesis. Specific tumor-immune interactions create the conditions necessary for tumor promotion. And the immunoregulatory factors produced by cancer cells play the role of mediators of this interaction (26, 27) (**Figure 1**).

This manuscript aims to focus mainly on the effects of factors produced by cancer cells, considering them separately (as far as possible) from the effects of the same factors but produced by other cells. Not all of these factors are produced by all tumors, but tumors always seem to produce at least some of them. Many tumor-produced factors are cytokines, but not all of them - for example, adenosine and kynurenines are not usually considered cytokines. Some of these factors are not even substances in the usual sense (e.g., reactive oxygen species (ROS) and reactive nitrogen species (RNS), so factors may be a better term.

In the following sections, we will examine the role of a number of factors produced by tumor cells in immunosuppression. In the conclusion we will discuss strategies of targeting the immunoregulatory factors emphasizing the importance of limiting their production by cancer cells.

## Tumor-produced factors and their role in cancer

Tumor-produced factors can be classified into various categories, including cytokines, chemokines, growth factors, and small molecule mediators. We will focus on several prominent examples from these groups.

## Cytokines

### IL-6

IL-6 in one of the key cytokines produced by normal cells during inflammation; besides, IL-6 is one of the most important cytokines in the tumor microenvironment - its protumor and anti-tumor effects are well known and extensively described in the literature (19, 24, 45). IL-6 is also produced by various cancer cell lines - human ovarian carcinoma cells (46), esophageal squamous cell carcinoma, cervical adenocarcinoma (6), and many others - at significantly lower levels than during acute inflammation, but in a constant manner.

In contrast to acute stimulation, prolonged stimulation by IL-6 and other cytokines activates a suppressive phenotype of myeloid cell lineages - MDSCs (47). In addition, IL-6 and IL-8 produced by tumor cells have been shown to play immunosuppressive roles by impairing the activity and function of natural killer cells (NK cells) (48). IL-6 exerts various other pro-cancer effects, being involved in angiogenesis (46) and acting as a growth factor for various types of cancer cells, including prostate cancer cells, breast cancer cells, esophageal adenocarcinoma cells, and others (49–51). Blocking the receptors for IL-6 results in anti-proliferative effects on cancer cells. For example, tocilizumab (an IL-6R-targeting antibody) decreased the proliferation of non-small cell lung cancer cell lines with an inhibition rate comparable to that of the typical anticancer drugs methotrexate and 5-fluorouracil (52). In another study, tocilizumab treatment decreased proliferation and invasion of osteosarcoma cell lines (143B, HOS, and Saos-2). In contrast, treatment with recombinant human IL-6 increased the proliferation of 143B and HOS cells (53).

IL-6 activates STAT3 which is a downstream transcription factor for IL-6, playing a major role in the process of MDSCs accumulation and acquisition of their immunosuppressive phenotype (48, 54). Moreover, it has been shown that IL-6 can induce arginase-1 expression in alternatively activated macrophages in STAT3 dependent manner, which can suppress CD4+ T cell proliferation (54).

### TNF

TNF (tumor necrosis factor) is expressed by a variety of cells, including tumor cells (55). For example, H. pylori-secreted TNF-inducing protein (Tipα) plays a role in increasing TNF levels in preneoplastic lesions detected in H. pylori-positive gastric lesions (56).

Low, sustained TNF production can induce an immunosuppressive phenotype through several mechanisms. Cancer cells can produce CCL2 and other chemokines in response to TNF stimulation, which enhances their metastatic potential (57) and recruit leukocytes with pro-metastatic effects to the tumor microenvironment (58).

TNF exerts its biological activity through several signaling pathways, including NF-κB and c-Jun N-terminal kinase (JNK). NF-κB mainly serves as an anti-apoptotic signal and JNK mediates the pro-apoptotic effect of TNF on cancer cells (57, 59). TNF has been shown to upregulate TAZ, a transcriptional co-activator that promotes self-renewal of breast cancer stem-like cells through the non-canonical NF-κB pathway (60). One such mechanism is the generation of ROS and RNS, which can induce DNA damage.

### IL-33

IL-33, an alarmin cytokine of the IL-1 family (61), is produced by various cells, including cancer cells (62, 63). It is crucial for the tumorigenic capacity of tumor-initiating cells (TICs), also known as cancer stem cells, which drive cancer progression and resistant to treatment (64, 65).

IL-33 attracts tumor-associated macrophages (TAMs) which express the IL-33 receptor ST2 and the high-affinity IgE receptor in close proximity to TICs (within a 50-mcm radius). TAMs create a high level of immunosuppressive TGF-β in the surrounding microenvironment. For instance, in squamous cell carcinoma model, IL-33 was found to be the most significantly upregulated cytokine in TGF-β-responsive TICs (65). IL-33 expression correlates with increased immunosuppressive macrophages, monocytes, and microglia in human glioma specimens and mouse models (66).

IL-33–ST2–NF-kB pathway stimulates paracrine TGF-β signaling to TICs, leading to further upregulation of IL-33 (65). Therefore, IL-33 production by cancer cells creates a positive feedback loop, increasing the number of immune cells with suppressive phenotypes and promoting drug-resistant cancer stem cells.

## Chemokines

### MCP-1/CCL2

Monocyte chemoattractant protein-1 (MCP-1/CCL2) was isolated in 1989 and found to be structurally identical to tumor cell-derived chemotactic factor (TDCF), responsible for tumor-associated macrophage (TAM) infiltration (67, 68). Many human cancer cells produce MCP-1, and it is found in cancer tissues such as glioma, meningioma, ovarian, lung, and breast cancers (67). MCP-1 levels are relatively low in many non-cancerous tissues with some exceptions, such as immune-privileged sites (67, 69). However, unstimulated stromal cells acquire the ability to produce MCP-1 under the influence of other tumor-produced factors (67).

In general, the level of MCP-1 is significantly associated with the accumulation of TAMs, which are known for their protumor effects (67). MCP-1 is crucial for establishing pre-metastatic niches and aiding cancer cell dissemination, with macrophages often involved in this process (70).

MCP-1 produced by cancer cells can attract macrophages and induce Wnt-1 upregulation, downregulating E-cadherin junctions in breast cancer cells and stimulating tumor cell dissemination (70). Additionally, MCP-1 binding to CCR2 on vascular endothelial cells directly stimulates angiogenesis (71).

### IP-10

Interferon gamma-induced protein 10 (IP-10), also known as CXC motif chemokine 10 (CXCL10), is a small cytokine-like protein produced by a wide variety of cell types. In healthy individuals, the expression of IP-10 is minimal, but it increases during the immune response due to stimulation by cytokine upregulation, especially by IFN-γ (72). The cells of several types of cancer (breast cancer, colon cancer, basal cell carcinoma, lung adenocarcinoma, etc.) are capable of producing IP-10, which can stimulate their growth, progression and metastasis in an autocrine manner (73).

IP-10 binds to the CXC chemokine receptor-3 (CXCR3) which is mainly expressed by T cells, NK cells, dendritic cells, macrophages, as well as some epithelial and cancer cells (73).

## Growth factors

Cancer cells are capable of producing various growth factors such as VEGF, TGF-β, PDGF, etc. As a result of the dysregulated autocrine and paracrine signaling networks in cancer, their role is mainly pro-tumor, stimulating epithelial-mesenchymal transition, angiogenesis, and immune suppression (74, 75).

### VEGF

Cancer cells are capable of producing VEGF to improve their own blood supply. According to the studies that evaluated the ability of tumor cells to produce various cytokines, VEGF is one of the most intensively produced cytokines by various tumor cells (76–78).

VEGF is a known factor that promotes cancer growth and metastasis by stimulating angiogenesis. In addition to stimulating angiogenesis, VEGF suppresses tumor immunity by inducing immunosuppressive cells such as tumor-associated macrophages, regulatory T cells (Treg), and MDSCs, and by inhibiting the maturation of dendritic cells (78).

VEGF suppresses immune responses by binding to its receptors (VEGFR1 and VEGFR2) on immune cells, activating the PI3K/Akt and MAPK pathways and contributing to CD8+ T cell exhaustion via expression of negative immune checkpoints, such as PD-1, CTLA-4, TIM-3 and others (79).

## Immune checkpoints

Tumor cells produce a multitude of ligands for immune checkpoints, which are presumed to play a pivotal role in the suppression of effector functions of the immune system. In addition to PD-L1/2, these ligands include galectin-3, galectin-9, and others (80).

### PD-L1

Due to advances in tumor immunotherapy, PD-L1 production is perhaps the first thing that comes to mind when we talk about immunosuppressive factors produced by tumors. Various types of immune cells are also capable of producing PD-L1, which is part of the autoregulation of the immune response, particularly during inflammation (81).

One of the major roles of PD-L1 produced by cancer cells is in many ways similar to the role of other tumor-derived factors - the orchestration of myeloid cells (M2 macrophages and others) that contribute to tumor infiltration, metastasis, and immune evasion (82). According to the recent study (22), tumor-derived PD-L1 does not directly protect tumor cells from cytotoxic T lymphocytes (CTL) cytotoxicity. Instead, tumor-derived PD-L1 promotes metastasis independent of primary tumor growth by suppressing inflammatory and CTL-driven responses within immunosuppressive niche, which are created through PD-L1 engagement with PD-1 on myeloid cells (22).

The molecular mechanism of this action of tumor-derived PD-L1 involves suppression of the intrinsic IFN-I-STAT1-CXCL9 pathway in myeloid cells through activation of the PD-1 protein-tyrosine phosphatase SHP-2 axis. This suppression, in turn, decreases CTL tumor infiltration in tumor metastases (22).

## Small molecule mediators

### Adenosine

Not all tumor-derived factors are cytokines. In particular, adenosine is a metabolic factor that is found in significant amounts in the typically hypoxic tumor microenvironment. Adenosine plays an important role in a variety of immunosuppressive and immunomodulatory mechanisms, culminating in the suppression of antitumor CD8+ T cell activity (83–86). Activation of adenosine receptors promotes the switch of macrophages to the anti-inflammatory M2 phenotype (87). In addition, adenosine attenuates the cytotoxic effect of NK-cells (mainly through A2 adenosine receptor signaling), leading to tumor immune escape in various tumors (84).

### Kynurenine

Kynurenine is the product of the degradation of tryptophan by indoleamine-2,3-dioxygenase (IDO) and tryptophan-2,3-dioxygenase (TDO). It has been demonstrated that metabolites of the kynurenine pathway can modify the behavior of immune cells, leading to a more tolerogenic phenotype (88). Kynurenine has been demonstrated to promote the expression of the protective TGF-β, the differentiation of Treg cells, and the induction of IDO1 expression in dendritic cells (DCs) (89–91). Kynurenine functions as an activating ligand for the aryl hydrocarbon receptor (AhR), a ligand-operated transcription factor. As an example, kynurenine induces an inflammatory positive autocrine feedback loop via the IDO1-AhR-IL-6-STAT3 signaling pathway, thereby enhancing tumor growth (88).

## Focus on the suppression of factors produced by cancer cells in the development of cancer treatment

In this article, we have examined only some of the factors secreted by tumor cells and capable of immunosuppression. However, there are many more such factors and their production can be considered as a phenomenon of chronic inflammation at the level of tumor cells. Apparently, there are no exclusively pro-tumor cytokines and other immunoregulatory factors (IRFs), but it can be assumed that their production by tumor cells is always unfavorable and is a potential target for antitumor therapy. The main essence of the proposed focus on targeting IRFs is to suppress their production by cancer cells.

The clinical application of targeting the IRFs produced by tumors involves several strategies: 1. direct neutralization of IRFs with specific antibodies; 2. IRFs receptor blockade; 3. inhibition of increased IRFs production by cancer cells. Many clinical trials are performed using the first two strategies. The use of these strategies usually leads to serious side effects due to systemic effects, as the action of all IRFs of a specific type is blocked, not just those produced by the tumor. For instance, immunotherapy with immune checkpoint inhibitors (anti-PD1, anti-PD-L1, etc.) which have radically changed the outcome of some cancers, cause strong autoimmune side effects that limit their use (45). In addition, there is a big variability in patient responses, and in most cases, patients do not respond to immune checkpoint immunotherapy (92).

However, the third strategy looks promising, since it may allow us to focus not on all factors of a certain type, but only on those produced by tumor cells. Indeed, the factors listed are not essentially tumor-specific but rather common factors released by many cell types during inflammation. Nevertheless, there is a theoretical possibility of interfering with the aforementioned IRFs in a manner that minimizes adverse effects. This can be achieved by focusing on the reduction of tumor cell IRFs production capabilities. Among other things, cancer cells differ from normal cells by overactivation of various signaling pathways. Blocking only one pathway may result in adaptive activation of signaling through other pathways, depending on individual patient characteristics. There are many targets in these signaling pathways, and simultaneous targeting of many of them is promising as it may reduce the production of IRFs by cancer cells.

But what kind of drugs can suppress a wide array of hyperactivated pathways in cancer cells to suppress the production of various IRFs? One example of this approach is using of multi-target drugs like multi-kinase inhibitors (93). By interacting with various intracellular signaling pathways, agents like multi-kinase inhibitors can block the production of IRFs by cancer cells. For instance, lenvatinib, a multi-target tyrosine kinase inhibitor suppresses VEGF production by hepatocellular carcinoma (HCC) cells (94, 95). Another multi-kinase inhibitor, Tivozanib, mediates immune modulation and reversal of tumor-induced immune suppression which correlates with survival of patients with cancer (96).

Some substances that have demonstrated potent antitumor effects *in vivo* have been observed to inhibit the production of various cytokines by tumor cells (for example, the polyphenolic metabolite of the intestinal microbiota urolithin A) (76, 97, 98). However, clinical studies of some of these compounds in cancer have yet to be conducted.

In the next section, we will briefly discuss the results of some clinical trials of the above-mentioned strategies, targeting the IRFs.

## Clinical limitations and challenges

Regarding the clinical applicability of targeting IRFs, there are certain limitations and challenges, including variability in patient responses and potential side effects. For instance, IL-6 signaling is involved in immunotherapy resistance (45, 99). This has been taken into account, and there are currently approximately 20 clinical trials evaluating the combination of IL-6 family antibodies and immune checkpoint inhibitors, showing variable patient responses (45). For example, in patients with advanced pancreatic cancer (NCT02767557), the addition of tocilizumab (an anti-IL-6R antibody) to gemcitabine/nab-paclitaxel did not result in improved overall survival rate at 6 and 24 months, although more patients were alive at 18 months in the gemcitabine/nab-paclitaxel/tocilizumab group (100). In newly diagnosed acute myeloid leukemia patients (NCT04547062) tocilizumab in combination with standard induction chemotherapy was considered to be safe and effective (1-year overall survival (OS) was estimated at 43% (21–88%) (101). Generally, the primary adverse effects of anti-IL-6/IL-6R antibodies are associated with bacterial infections (45).

A number of clinical studies have analyzed the therapeutic value of TNF-TNFR antagonists in cancer treatment. Some phase I and II trials showed disease stabilization in various malignancies, and the phase Ib trial (NCT03293784) combining TNF inhibitor certolizumab with anti-PD-1/anti-CTLA-4 in melanoma patients demonstrated safety and high response rates (58, 102). The recent trial of TNF-α inhibitor certolizumab plus chemotherapy in stage IV lung adenocarcinomas is notable for targeting cancer-induced inflammation involving tumor-produced IRFs. It aimed to disrupt the paracrine inflammatory loop, where chemotherapy-induced cytotoxic stress leads to TNF-α secretion by endothelial cells, promoting cancer-cell production of CXCL1/2 and recruitment of MDSCs. The median response duration was 9.0 months (range 5.9 to 42.6 months). This study shows strong pharmacodynamic inhibition of cytokines in the paracrine inflammatory loop (103).

A recent meta-analysis showed that VEGF/VEGFR inhibitors combined with chemotherapy improved outcomes in platinum-resistant ovarian cancer compared to monotherapy. This combination therapy caused more side effects like hypertension, mucositis, proteinuria, and diarrhea, than monotherapy, however, these side effects were manageable and well-tolerated (104). Inhibiting VEGFR-related pathways with kinase inhibitors might be more effective because these inhibitors often target multiple cancer-promoting signaling pathways simultaneously (105). Another meta-analysis compared the efficacy and safety of two first-line therapies for unresectable hepatocellular carcinoma: anti-PD-1/L1 antibody plus anti-VEGF antibody, and anti-PD-1/L1 antibody plus VEGFR-targeted tyrosine kinase inhibitor. The anti-PD-1/L1 and anti-VEGF combination showed the longest overall survival (OS), while the anti-PD-1/L1 and VEGFR-targeted tyrosine kinase inhibitor combination provided better progression-free survival (PFS) but with lower safety (106).

As mentioned, adenosine, a tumor-produced IRF, is a promising target with at least 54 active clinical trials (107). A first-in-human study of adenosine 2A and 2B receptor antagonists in advanced solid tumors (NCT04969315) recently began, with no serious adverse events or dose-limiting toxicities observed so far (108). The phase I clinical trial of ciforadenant, a small-molecule adenosine 2A receptor antagonist, in patients with renal cell cancer showed clinical responses both alone and in combination with an anti-PD-L1 antibody, including in subjects who had progressed on PD-1/PD-L1 inhibitors. The estimated OS exceeded 90% at 25 months for the combination group (ciforadenant plus the PD-L1 inhibitor atezolizumab). Ciforadenant efficacy was associated with CD8+ T cell tumor infiltration and diversification of TCR repertoire (109).

Despite the above-mentioned positive changes, their magnitude is usually far from 100%. The reason for this may be that above-mentioned therapies usually target only one factor, whereas many factors are involved in tumorigenesis and cancer-related inflammation (110). As an example, preclinical studies show that while potent anti-angiogenic agents can suppress tumor-induced neovascularization, cancer cells often adapt by increasing invasiveness and metastasis (105). The third strategy, which involves the inhibition of IRFs production with multi-target drugs, appears to be promising. The recent phase I study of tinengotinib, a multiple kinase inhibitor, as a single agent in patients with advanced solid tumors showed that tinengotinib was well tolerated and indicated potential clinical benefit in FGFR inhibitor-refractory cholangiocarcinoma, HER2-negative breast cancer (including triple-negative breast cancer), and castration-resistant prostate cancer. A total of 13 patients (30.2%) achieved partial response or stable disease (111). Another recent phase I study of KC1036, a multiple kinase inhibitor, as a single agent in heavily pre-treated patients with advanced solid tumors revealed a manageable safety profile and preliminary antitumor activity. Among 36 patients who had at least one efficacy evaluation disease control rate (DCR) was 80.6% (112). It is noteworthy that the two aforementioned studies (111, 112) exhibited a shared adverse effect: hypertension. Phase II trial of KC1036 showed its promising anti-tumor activity in patients with previously treated advanced esophageal squamous cell carcinoma (the DCR was 83.3%) (113).

## Conclusion and prospects

Many of the clinical trials mentioned were conducted under unfavorable conditions, with patients in advanced stages of disease and having undergone multiple therapies that compromised their immune system (58, 114, 115). Additionally, most trials focused on suppressing a single IRF. Targeting multiple factors produced by tumors, especially early in treatment, might be more effective. This could be achieved with agents that modulate various intracellular signaling pathways, such as multi-kinase inhibitors, which have a relatively favorable safety profile and potential as disease-modifying cancer therapies (116–118). It should be noted that the inhibition of IRFs production is not the sole mechanism of action of such agents; however, it may be of particular significance in the context of limiting cancer-induced immune suppression. A focus on the capacity of multi-target drugs to suppress IRFs production may assist in the identification of the most promising drugs for clinical trials.
